# European Expert Opinion on Infrarenal Sealing Zone Definition and Management in Endovascular Aortic Repair Patients: A Delphi Consensus

**DOI:** 10.1177/15266028221082006

**Published:** 2022-03-17

**Authors:** Jean-Paul P. M. de Vries, Roy Zuidema, Colin D. Bicknell, Robert Fisher, Mauro Gargiulo, Nicolas Louis, Kyriakos Oikonomou, Giovanni Pratesi, Michel M. P. J. Reijnen, Andrés Reyes Valdivia, Vincent Riambau, François Saucy

**Affiliations:** 1 Department of Surgery, Division of Vascular Surgery, University Medical Centre Groningen, Groningen, The Netherlands; 2 Department of Surgery and Cancer, Imperial College London and Imperial Vascular Unit, Imperial College Healthcare NHS Trust, London, UK; 3 Department of Vascular Surgery, Liverpool University Hospitals NHS Foundation Trust, Liverpool, Merseyside, UK; 4 Vascular Surgery, Department of Experimental, Diagnostic and Specialty Medicine, University of Bologna, Bologna, Italy; 5 Chirurgie vasculaire, Clinique Les Franciscaines, Nîmes, France; 6 Department of Vascular Surgery, University Medical Center Regensburg, Regensburg, Germany; 7 Clinic of Vascular and Endovascular Surgery, Ospedale Policlinico San Martino, Department of Integrated Surgical and Diagnostic Sciences, University of Genoa, Genoa, Italy; 8 Department of Vascular Surgery, Rijnstate Hospital, Arnhem, The Netherlands; 9 Multi-Modality Medical Imaging Group, University of Twente, Enschede, The Netherlands; 10 Department of Vascular and Endovascular Surgery, Ramón y Cajal’s University Hospital, Madrid, Spain; 11 Vascular Surgery Division, CardioVascular Institute, Hospital Clinic, Barcelona, Spain; 12 Service of Vascular Surgery, Etablissement Hospitalier de la Côte, Morges, Switzerland; * Department of Vascular and Endovascular Surgery, Cardiovascular Surgery Clinic, University Hospital Frankfurt and Johann Wolfgang Goethe University Frankfurt, Frankfurt, Germany

**Keywords:** sealing zone, EVAR, AAA, Delphi consensus

## Abstract

**Purpose:**

The purpose of the study was to provide a consensus definition of the infrarenal sealing zone and develop an algorithm to determine when and if adjunctive procedure(s) or reintervention should be considered in managing patients undergoing endovascular aortic repair (EVAR) for infrarenal abdominal aortic aneurysm (AAA).

**Methods:**

A European Advisory Board (AB), made up of 11 vascular surgeons with expertise in EVAR for AAA, was assembled to share their opinion regarding the definition of preoperative and postoperative infrarenal sealing zone. Information on their current clinical practice and level of agreement on proposed reintervention paths was used to develop an algorithm. The process included 2 virtual meetings and 2 rounds of online surveys completed by the AB (Delphi method). Consensus was defined as reached when ≥ 8 of 11 (73%) respondents agreed or were neutral.

**Results:**

The AB reached complete consensus on definitions and measurement of the pre-EVAR target anticipated sealing zone (TASZ) and the post-EVAR real achieved sealing zone (RASZ), namely, the shortest length between the proximal and distal reference points as defined by the AB, in case of patients with challenging anatomies. Also, agreement was achieved on a list of 4 anatomic parameters and 3 prosthesis-/procedure-related parameters, considered to have the most significant impact on preoperative and postoperative sealing zones. Furthermore, the agreement was reached that in the presence of visible neck-related complications, both adjunctive procedure(s) and reintervention should be contemplated (100% consensus). In addition, adjunctive procedure(s) or reintervention can be considered in the following cases (% consensus): insufficient sealing zone on completion imaging (91%) or on the first postoperative computed tomography (CT) scan (91%), suboptimal sealing zone on completion imaging (73%) or postoperative CT scan (82%), and negative evolution of the actual sealing zone over time (91%), even in the absence of visible complications.

**Conclusions:**

AB members agreed on definitions of the pre- and post-EVAR infrarenal sealing zone, as well as factors of influence. Furthermore, a clinical decision algorithm was proposed to determine the timing and necessity of adjunctive procedure(s) and reinterventions.

## Introduction

After 3 decades of endovascular aneurysm repair (EVAR) for abdominal aortic aneurysms (AAA), the infrarenal neck is considered the most important determining factor for an uncomplicated and sustainable outcome. As the envelope has been pushed toward treating more challenging infrarenal necks with standard endografts, there are numerous publications regarding hostile aortic neck criteria.^1–4^ During preoperative planning and sizing, aortic neck length, diameter, suprarenal and infrarenal angulation, shape, and occurrence of calcium and thrombus are measured by most endovascular specialists, using dedicated software. Moreover, all endograft manufacturers have defined specific instructions for use (IFU) concerning infrarenal neck characteristics. Infrarenal neck length seems to be one of the most important criteria to consider, with a minimum of 10 or 15 mm, according to the IFU of most commercially available endografts.^3^

According to most CoreLab definitions, the infrarenal neck ends when the aortic diameter increases >10% compared with baseline (ie, the diameter just below the lower margin of the lowest renal artery).^5,6^ Although the determination of the pre-EVAR neck characteristics gives the treating physicians a handle in the sizing and planning process, it does not always match with the actual circumferential seal of the endograft in the aortic neck after deployment. Oversizing the endograft’s main body often extends the seal compared with the predefined aortic neck length. However, especially in hostile necks, this does not always have the anticipated and desired effect.^7^ It seems reasonable to assume that the post-EVAR achieved circumferential apposition between the endograft and the aortic wall is a better indicator for outcome than the pre-EVAR determined aortic neck characteristics alone.

The so-called sealing zone in the infrarenal aortic neck has received less attention in EVAR literature so far. A possible explanation for this might be that it is harder to define than the well-known aortic neck criteria. It also depends on the positioning of the endograft during the procedure. Moreover, the circumferential apposition between the endograft and the aortic wall has to be determined on the post-EVAR computed tomography (CT) scan, which is not a standard measurement so far.^8^ A Delphi method is often used to orchestrate expert opinions systematically when evidence is scarce or lacking, and research questions cannot simply be studied with experimental and epidemiological methods.^9^ In this study, the Delphi method is used to propose a consensus definition of the infrarenal sealing zone. Furthermore, it provides an algorithm to determine when and if adjunctive procedure(s) or reintervention should be considered in case of potential proximal sealing failure of the endograft.

## Method

An independent Advisory Board (AB), made up of 11 European vascular surgeons with extensive experience in EVAR for AAA, was gathered. Efforts were made to be as inclusive as possible to represent geographical variety. Seven European countries were represented in the AB: France, Germany, Italy, The Netherlands, Spain, Switzerland, and the United Kingdom. The Delphi methodology was applied to share their opinion on the definition of the infrarenal sealing zone and its impact on considering adjunctive procedure(s) and reintervention for patients undergoing EVAR for infrarenal AAA. A 4-step approach was implemented:

*Step 1*: A literature review on currently available evidence on the sealing zone concept was conducted. PubMed and EMBASE databases were searched from January 2010 to October 2020 using the following search strategy: (((infrarenal) adj2 (aort*-aneurysm* or AAA)) and ((sealing or landing or apposition) adj5 (zone* or area*)) and ((zone* or area*) adj5 (definition* or measurement* or length* or long or ‘mm’))). A total of 30 publications reporting the definition of the sealing zone and its measurement method were selected and used as a base for developing the first round of the questionnaire.
*Step 2*: A Web-based Delphi panel process, a method used in literature to determine and integrate experts’ opinions on a particular topic and attempt to reach a consensus by using consecutive rounds of survey questions, was conducted to develop consensus recommendations.^9^ The Delphi panel comprised 2 online questionnaire rounds via Qualtrics (Qualtrics, Provo, Utah).^10^ In line with previous reports reporting consensus threshold to vary from 70% to 80%,^11^ the AB members defined that consensus was reached when ≥73% of respondents agreed with or were neutral on the question response (ie, at least 8 of 11 AB members). For most of the questions, AB members could add comments. The first round of the online survey was sent to all AB members before the first virtual meeting to establish the initial level of agreement.
*Step 3*: The results of the first survey round were analyzed and debated by the AB members in a meeting held in March 2021. By doing so, AB members were encouraged to reflect on their initial answers before selecting a response in the next round. Specifically, the following topics were discussed in detail by the AB members:
  - Infrarenal sealing zone definition and measurement methods.
  - Parameters of influence on the sealing zone, considering both patient’s objective anatomic characteristics and prosthesis-/procedure-related factors.
  - Scenarios in which intraoperative adjunctive procedure(s) and reintervention during follow-up may be considered. Based on the achieved postoperative sealing zone and presence of proximal sealing zone complications, such as type Ia endoleaks or migration ≥10 mm.

The AB members decided to slightly modify some questions to clarify their meaning and add some questions in the second survey round to deepen some topics.

*Step 4*: Based on the Delphi method described above, the second survey round was sent to all AB members to appraise the new level of agreement after the meeting. A second virtual meeting was held in April 2021 to present and discuss the results of the second survey round and draft the intervention algorithm based on the AB responses.

### Statistical Analysis

To summarize responder characteristics and survey results, data are expressed as median (Q1, Q3) for continuous data or as fractions and percentages for categorical data. Excel Office 365 (Microsoft, Redmond, Washington) was used to perform analyses.

## Results

All of the 11 AB members completed both rounds of the online survey. The median number of annually performed open and endovascular procedures for AAA for all members was 50 (45, 90), with a median of 85% (63%, 85%) among them performed by endovascular repair. The first survey questions addressed the AB opinion on the current level of evidence on pre-EVAR and post-EVAR sealing zone definitions, and their relevance to treatment outcomes and patients management strategies, respectively (Table 1). All AB members (11/11, 100%) agreed that although the pre-EVAR sealing zone assessment is highly relevant in terms of procedural outcomes, the available evidence on its definition and measurement is lacking. Similarly, according to most responders (10/11, 91%), the post-EVAR sealing zone evaluation significantly affects patient follow-up management. However, they consider this inconclusive, due to the low level of evidence (Table 1). As a good agreement level was noted at the first survey round on these preliminary questions, they were not included in the second round.

**Table 1.** Survey Introduction Results.

Survey questions	n (%)
In the last 3 years, what is the average number of open and endovascular procedures/year you performed for AAA?	
0–30 procedures/year	2 (18)
31–60 procedures/year	4 (36)
61–90 procedures/year	2 (18)
>90 procedures/year	3 (27)
In the last 3 years what is the percentage of endovascular repair among all the procedures you performed for AAA?	
0%–25%	0 (0)
26%–50%	0 (0)
51%–75%	6 (55)
76%–100%	5 (45)
In your opinion, the scientific literature currently available relating to the *preoperative* sealing zone definition and measurement is adequate to identify the best evidence-based intervention.	
Agree	0 (0)
Neutral	2 (18)
Disagree	9 (82)
In your opinion, the scientific literature currently available relating to the *postoperative* sealing zone definition and measurement is adequate to identify the best evidence-based intervention.	
Agree	1 (9)
Neutral	1 (9)
Disagree	9 (82)
I consider the *preoperative* sealing zone evaluation relevant for the treatment outcomes.	
Agree	11 (100)
Neutral	0 (0)
Disagree	0 (0)
I consider the *postoperative* sealing zone evaluation relevant for the patient’s management strategy (eg, follow-up frequency and length, CT scans/year).	
Agree	10 (91)
Neutral	0 (0)
Disagree	1 (9)

Abbreviations: AAA, abdominal aortic aneurysm; CT, computed tomography.

### Sealing Zone Definition and Measurement

**Figure 1.**
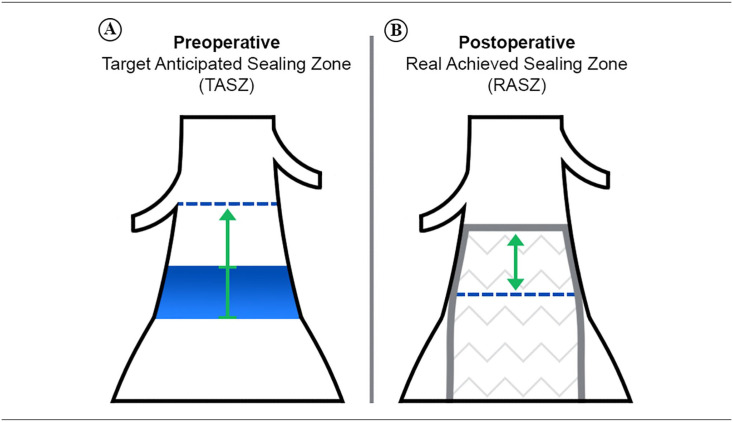
Schematic overview of (A) the pre-EVAR target anticipated sealing zone (TASZ) and (B) the post-EVAR real achieved sealing zone (RASZ) in the infrarenal aorta. The TASZ is the length starting just inferior to the distal renal artery and ending at the most proximal slice at which the endograft is anticipated to no longer be in circumferential apposition to the aortic wall (also considering the endograft oversizing). The distal point of reference (blue area) is influenced by the degree of oversizing. The RASZ is the length starting at the proximal end of the endograft fabric and ending where the endograft material is no longer circumferentially apposed to the aortic wall. The TASZ and RASZ should be measured over the centerline between the reference points, or in case of patients with challenging anatomies, as the shortest length between the reference points.

During the first round of the survey, AB members were asked to select, among the proposed sealing zone definitions reported in the literature, the one they deemed most appropriate. As none reached the consensus threshold (Table 2), AB members drew their own definition based on their clinical experience. Members agreed to the need to differentiate the definitions of the pre-EVAR/optimal sealing zone (ie, target anticipated sealing zone [TASZ]) and the post-EVAR/actual sealing zone (ie, real achieved sealing zone [RASZ]); therefore, the question was split into 2 questions in the second survey round. They also highlighted the importance of referring to stent graft oversizing in both definitions, considering its impact on sealing and fixation.^12^ In the second round of the survey, the proposed definitions of TASZ and RASZ reached 100% consensus (Table 2). A schematic overview of the TASZ and RASZ is shown in Figure 1.

**Table 2.** First and Second Survey Rounds Results on Preoperative and Postoperative Infrarenal Sealing Zone Definition and Measurement.

First round of survey		Second round of survey	
Survey question	n (%)	Survey question	n (%)
Based on your experience, which do you think is the most appropriate definition of “proximal sealing zone” among those reported in literature?		I consider the most appropriate definition of the preoperative *Target Anticipated sealing zone* as: “Length starting just inferior to the distal renal artery and ending at the most proximal slice at which the endograft is *anticipated* to no longer be in proper apposition to the aortic wall (also considering the endograft oversizing).” • Agree • Neutral • Disagree	10 (91) 1 (9) 0 (0)
1. Distance from the proximal end of the endograft fabric to the beginning of the aneurysm	1 (9)		
2. Length over which the endograft material is circumferentially in contact with the aortic wall	4 (36)		
3. Length between the proximal end of the endograft fabric and the most proximal slice where circumferential apposition of the fabric to the aortic neck is lost	0 (0)		
4. Length starting just inferior to the distal renal artery and ending at the most proximal slice at which the endograft is no longer in complete 360° apposition to the aortic wall	4 (36)	I consider the most appropriate definition of the postoperative *Real Achieved sealing zone* as: “Length starting at the proximal end of the endograft fabric and over which the endograft material is in proper apposition to the aortic wall (also considering the endograft oversizing).” • Agree • Neutral • Disagree	10 (91) 1 (9) 0 (0)
5. Length of the aortic neck covered by the endograft	0 (0)		
6. Aortic segment proximal to the AAA that is normal, with parallel walls and without significant calcification or mural thrombus	2 (18)		
7. Other	0 (0)		
Based on your experience, which is the most appropriate method for measuring the *preoperative* sealing zone?		In case of patients with challenging anatomies, I consider the most appropriate method for measuring the preoperative *Target Anticipated sealing zone* as: “The *shortest length* between two orthogonal boundary planes that include a proximal and a distal point of reference.” • Agree • Neutral • Disagree	10 (91) 1 (9) 0 (0)
1. Length over the centerline between 2 orthogonal boundary planes that include a proximal and a distal point of reference	8 (73)		
2. Length over the inner curvature between 2 orthogonal boundary planes that include a proximal and a distal point of reference	2 (18)		
3. Length over the outer curvature between 2 orthogonal boundary planes that include a proximal and a distal point of reference	0 (0)		
4. Distance over the arterial wall between two 3D coordinates that are located on the arterial wall	1 (9)		
Based on your experience, which is the most appropriate method for measuring the *postoperative* sealing zone?		In case of patients with challenging anatomies, I consider the most appropriate method for measuring the postoperative *Real Achieved sealing zone* as: “The *shortest length* between two orthogonal boundary planes that include a proximal and a distal point of reference.” • Agree • Neutral • Disagree	10 (91) 1 (9) 0 (0)
1. Length over the centerline between 2 orthogonal boundary planes that include a proximal and a distal point of reference	7 (64)		
2. Length over the inner curvature between 2 orthogonal boundary planes that include a proximal and a distal point of reference	3 (27)		
3. Length over the outer curvature between 2 orthogonal boundary planes that include a proximal and a distal point of reference	0 (0)		
4. Distance over the arterial wall between two 3D coordinates that are located on the arterial wall	1 (9)		
Based on your experience, what image you usually consider for measuring the sealing zone?		The measurement of the *Target Anticipated sealing zone* by preoperative CT scan should be recommended. • Agree • Neutral • Disagree	11 (100) 0 (0) 0 (0)
1. Preoperative CT scan (or MRA, if CT is not feasible or recommended) evaluated using a 3D workstation	5 (45)		
2. First postoperative CT scan (or MRA, if CT is not feasible or recommended) evaluated using a 3D workstation	0 (0)		
3. Both preoperative CT and first postoperative scan (or MRA, if CT is not feasible or recommended) evaluated using a 3D workstation	5 (45)	The measurement of the *Real Achieved sealing zone* by postoperative CT scan should be recommended. • Agree • Neutral • Disagree	9 (82) 2 (18) 0 (0)
4. Other (ie, first postoperative and 1-year CT scans)	1 (9)		

Abbreviations: 3D, 3-dimensional; AAA, abdominal aortic aneurysm; CT, computed tomography; MRA, magnetic resonance angiography.

Concerning the measurement method, questions in the first survey round aimed to assess whether AB members solely evaluate the center lumen line (CLL) length or if inner and outer aortic curves have any role in the pre- and post-EVAR sealing zone measurement. Although members expressed a preference for the CLL measurement, they suggested that in case of a high degree of infrarenal aortic angulation and/or challenging pre-EVAR aortic neck anatomies, both TASZ and RASZ measurements should be based on the shortest length between the predefined reference points mainly because the CLL length might overestimate the sealing zone in this case. The 2 amended definitions reached 100% consensus (Table 2). When asked which imaging technique(s) the AB members use in their clinical practice for measuring the infrarenal sealing zone, half of the responders answered that they evaluate both the pre- and post-EVAR sealing zone by CT scans, while the other half merely measure the TASZ through preoperative CT scan. During the first virtual meeting, a subsequent discussion revealed that members agree on the importance of recommending the postoperative evaluation for confirmation of the RASZ and as a prognostic indicator. The question was also repeated for TASZ and RASZ and reached 100% consensus in the second round of the survey (Table 2).

### Sealing Zone Parameters

In the first survey round, the whole board (11/11, 100%) agreed that both patient’s objective anatomic characteristics and prosthesis-/procedure-related parameters affect the infrarenal sealing zone. Specifically, 6 members (55%) stated that they have the same weight, whereas the remaining 5 members (45%) answered that the anatomic parameters have more impact than the prosthesis-/procedure-related ones.

**Figure 2.**
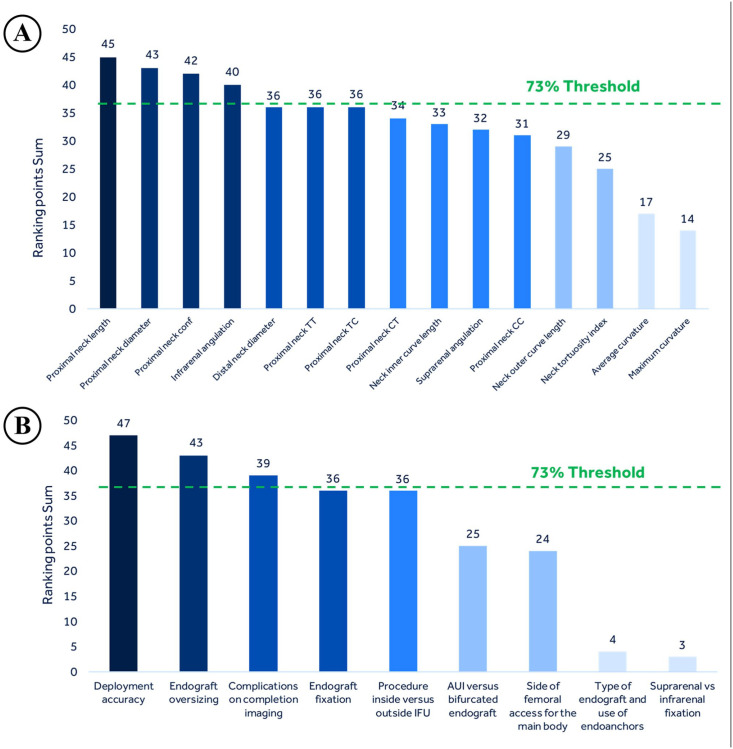
Survey results on parameters influencing the preoperative target anticipated sealing zone (A) and the postoperative real achieved sealing zone (B). Conf: configuration (conical vs non-conical), TT, thrombus thickness; TC, thrombus circumference; CT, calcification thickness; CC, calcification circumference; IFU, instructions for use; AUI, aortouniiliac.

When AB members were asked which anatomy-related parameters they consider as the most relevant for the preoperative sealing zone, the consensus was achieved for 4 parameters: “proximal aortic neck length (CLL length)” (89%), “proximal aortic neck diameter (immediately below the lowest renal artery)” (85%), “proximal aortic neck configuration (conical versus non-conical)” (83%), and “infrarenal angulation” (79%). During the first meeting, the AB members agreed that thrombus-related parameters are more relevant to the sealing zone than the calcium-related ones (Figure 2A). AB consensus was reached on 3 prosthesis- and procedure-related parameters that were considered to have the most significant impact on the postoperative sealing zone: “deployment accuracy of the endograft (optimal vs suboptimal)” (93%), “endograft oversizing (proximal graft diameter/proximal neck diameter)” (85%), and “presence of endograft complications (eg, endoleak/kinking/stenosis) on completion imaging” (77%) (Figure 2B). In the second survey round, these 2 questions were modified to measure the AB consensus on the 4 selected anatomic and the 3 selected procedure-/prosthesis-related parameters. Their answers reflected broad agreement with the parameter’s selection done in the first round: 91% (10/11) and 100% (11/11), respectively, with no neutral answers. Furthermore, during the first meeting, the AB agreed to include 2 questions in the second survey round to investigate the appropriate range and measurement of proximal endograft oversizing. Second survey round results showed that the board agreed in considering 10% to 30% as the appropriate range for the infrarenal oversizing (100% consensus, one neutral answer). Moreover, both maximum and average aortic neck diameters should be taken for infrarenal oversizing measurement in patients with challenging anatomies (eg, conical neck) (91% consensus, one neutral answer).

### Algorithm Development

One of the project’s main objectives was to develop an algorithm to aid the clinical decision-making process to determine when to consider intraoperative adjunctive procedure(s) or reintervention in patients treated with EVAR for infrarenal AAA. Table 3 summarizes the results of the 2 survey rounds in terms of consensus on the algorithm. Although, in the second round of the survey, the agreement was reached for all the questions addressed in the algorithm, in 4 of them the percentage of neutral respondents was crucial to get the consensus threshold of 73%.

**Table 3.** First and Second Survey Rounds Results in Terms of Agreement With the Proposed Algorithm.

First round of survey		Second round of survey		
Survey question	n (%)	Survey question	n (%)	Reasons for disagreement
I consider reintervention necessary in case of presence of complications (eg, type I endoleak) on completion imaging. • Agree • Neutral • Disagree	8 (73) 2 (18) 1 (9)	I consider adjunctive procedure(s) in case of presence of neck-related complications (eg, type I endoleak) on completion imaging. • Agree • Neutral • Disagree *If you agree, specify*: Is the presence of these complications *alone* enough to perform adjunctive procedure(s)? • Agree • Neutral • Disagree	11 (100) 0 (0) 0 (0) 8 (73) 3 (27) 0 (0)	None
I consider reintervention necessary in case of presence of complications (eg, type I endoleak) on 1-month follow-up imaging. • Agree • Neutral • Disagree	9 (82) 2 (18) 0 (0)	I consider reintervention in case of presence of neck-related complications (eg, type I endoleak) on postoperative CT scan. • Agree • Neutral • Disagree *If you agree, specify*: Is the presence of these complications *alone* enough to perform reintervention? • Agree • Neutral • Disagree	11 (100) 0 (0) 0 (0) 8 (73) 2 (18) 1 (9)	None
I consider reintervention in case of insufficient/suboptimal actual sealing zone and absence of visible complications on completion imaging, to prevent any complications. • Agree • Neutral • Disagree	4 (36) 3 (27) 4 (36)	I consider adjunctive procedure(s) in case of *insufficient* sealing zone and absence of visible neck-related complications on *completion imaging*, to prevent any complications. • Agree • Neutral • Disagree	7 (64) 3 (27) 1 (9)	One AB member stated that—in case of absence of complications—his decision also depends on further elements (ie, age of patient)
		I consider adjunctive procedure(s) in case of *suboptimal* sealing zone and absence of visible neck-related complications on *completion imaging*, to prevent any complications. • Agree • Neutral • Disagree	6 (55) 2 (18) 3 (27)	One AB member stated that—in case of absence of complication—his decision also depends on further elements (ie, age of patient); others would recommend to wait and follow-up the seal over time.
		I consider reintervention in case of *insufficient* sealing zone and absence of visible neck-related complications on *postoperative CT scan*, to prevent any complications. • Agree • Neutral • Disagree	6 (55) 4 (36) 1 (9)	One AB member suggested to follow-up the sealing zone for 6 to 12 months and then evaluate reintervention.
		I consider reintervention in case of *suboptimal* sealing zone and absence of visible neck-related complications on *postoperative CT scan*, to prevent any complications. • Agree • Neutral • Disagree	4 (36) 5 (46) 2 (18)	AB members stated that, in the absence of complications, they would prefer to wait and follow-up the seal over time.
I consider reintervention in case of negative evolution of the actual sealing zone over time. • Agree • Neutral • Disagree	6 (55) 4 (36) 1 (9)	I consider reintervention in case of negative evolution of the actual sealing zone over time and absence of visible neck-related complications on follow-up CT scan(s), to prevent any complications. • Agree • Neutral • Disagree	9 (82) 1 (9) 1 (9)	One AB member stated that he would not consider reintervention in case of no complications and no increase of the AAA diameter.

Abbreviations: AAA, abdominal aortic aneurysm; AB, advisory board; CT, computed tomography.

**Figure 3.**
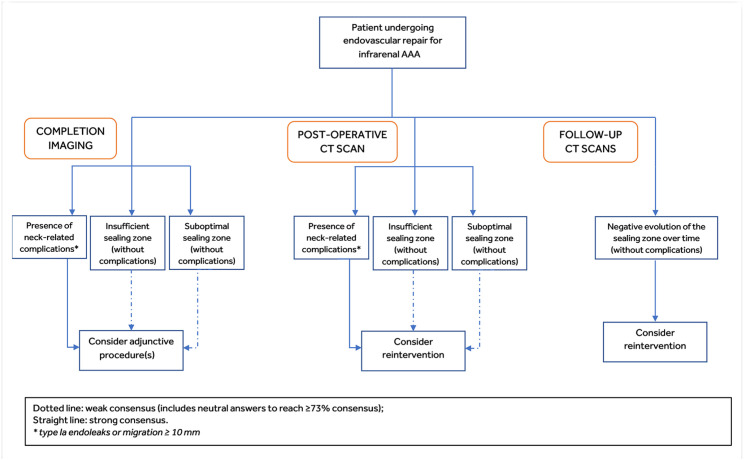
Algorithm developed by the Advisory Board. AAA, abdominal aortic aneurysm; CT, computed tomography.

In the first survey round, AB members were asked whether they consider it necessary to reintervene if either completion angiography or the 1-month imaging show complications such as type I endoleaks. Although consensus was reached during the first meeting discussion, it became apparent that these questions needed to be adjusted: AB suggested removing the word *necessary*, which indicates the procedure as “mandatory,” and replacing it with the term *consider*, to be interpreted as “contemplate the possibility to perform any reinterventions/adjunctive procedures.” Moreover, they agreed to establish “neck-related” complications to denote proximal sealing zone complications such as type Ia endoleaks or migration ≥10 mm. In all the question referring to the completion imaging (ie, intraoperative completion angiography), the word *reintervention* was replaced by “adjunctive procedure(s)” to indicate an intraoperative intervention rather than a reintervention. The second survey round results revealed a high level of agreement (100% consensus, with no neutral answers) on the fact that AB members consider adjunctive procedure(s)/reintervention if images show evidence of proximal sealing zone complications. In addition, they agreed to assess the presence of neck-related complications alone, enough to proceed with adjunctive procedure(s) (100% consensus) and reintervention (91% consensus). When asked whether members consider any reintervention in case of insufficient or suboptimal actual sealing zone and absence of visible complications on completion imaging, the consensus threshold was not reached in the first survey round. A subsequent discussion during the virtual meeting revealed that the question needed to be modified by distinguishing insufficient from the suboptimal postoperative sealing zone. The AB defined suboptimal achieved sealing zone as infrarenal sealing zone length with minimal deviation from device IFU requirements, and insufficient achieved sealing zone as infrarenal sealing zone length with significant deviation from device IFU requirements. Advisory Board members also agreed to further split the question to consider both the possibility of performing adjunctive procedure(s) following the completion imaging evaluation and reintervention based on the first postoperative CT scan assessment. In the second survey round, weak consensus (91% including 27% of neutral answers) was achieved on the question asking whether members would consider adjunctive procedure(s) in the event of insufficient RASZ and absence of visible neck-related complications on completion angiography. Consensus decreased to 73% (including 18% of neutral answers) for suboptimal RASZ. Especially in the case of suboptimal RASZ, some responders clarified they would prefer to modify the surveillance regimen instead of proceeding with additional procedures, or they would favor individualized treatment according to the patients’ life expectancy and follow-up adherence. Similar consensus values resulted from the second survey round questions focusing on the first postoperative CT scan assessment: 91% (including 36% of neutral answers) for insufficient RASZ with the absence of neck-related complications, and 82% (including 46% of neutral responses) for a suboptimal RASZ. Also, in this case members who disagreed explained their preference to wait and closely follow-up patients before eventually performing a reintervention. In line with the previous results, most responders agreed (82%) or were neutral (9%) in considering reintervention in case of a decrease in the RASZ over time detected on CT scan(s), even in the absence of visible neck-related complications. The final proposed algorithm is provided in Figure 3.

## Discussion

There seems no doubt that the infrarenal sealing zone concept is carving itself a niche in the world of EVAR. The consensus was reached by the AB members that CT-based measurement of the sealing zone is recommended. The current Delphi method shows that the term *sealing zone* should be differentiated between the pre-EVAR TASZ and the post-EVAR RASZ. To date, pre-EVAR sizing and planning are more or less finding a balanced choice with the well-known aortic neck characteristics (length, diameter, infrarenal angulation, and shape) and the endograft’s IFU as ingredients. The TASZ can differ positively from the CoreLab-defined preoperative neck length because of extra oversizing of the endograft or negatively due to unexpected setbacks during the procedure. In general, more oversizing will result in a larger TASZ. Still, the exact TASZ also depends on the complex geometry of the aortic neck and is therefore hard to predict with current imaging techniques.

It should come as no surprise that consensus was reached among the AB members concerning the most critical anatomical parameters of influence on TASZ: proximal aortic neck length, diameter, shape, and infrarenal angulation. Almost a decade ago, Antoniou et al published a landmark meta-analysis in which these anatomical characteristics were the main ingredients to define aortic necks, either as “friendly” or “hostile.”^1^ Patients with hostile necks had a 4-fold higher risk to develop a type Ia endoleak and a 9-fold higher risk of aneurysm-related mortality within 1-year post-EVAR. More recently, 9 experienced Italian vascular surgeons applied a Delphi consensus method demonstrating their expert opinion on hostile neck definitions.^13^ Besides the neck criteria mentioned above, they identified the presence of circumferential aortic neck calcification as a hostile neck characteristic. The Italian expert group was probably the first to rank a (minimum) combination of hostile neck criteria unfavorable for endovascular repair. Although it makes sense to hypothesize that a variety of hostile neck criteria will increase the risk for EVAR-related complications compared with a single neck criterion, robust evidence is lacking. The individual value of all hostile neck parameters and their relationship with each other is unclear. We need to implement new developments in imaging techniques and data processing to solve this issue in the upcoming years. To date, morphological shape features can be analyzed with the construction of statistical shape models, and machine learning may be used to associate these aortic neck shape features with post-EVAR complications. Liang et al described this technique to investigate the association between ascending aortic aneurysm shape features and finite element analysis-predicted rupture risk.^14^ This promising tool is, however, still in its infancy.

All but one AB member agreed that the most appropriate method for measuring the preoperative and postoperative sealing zone in patients with challenging anatomies is the shortest length between 2 orthogonal boundary planes that include a proximal and distal reference. Literature on sealing zone calculation is scarce and mainly focuses on the RASZ. Schuurmann et al systematically reviewed existing literature and found 3 methods.^8^ Basically, apposition of the endograft and the aortic wall can be measured based on CLL reconstructions, or by determining the shortest distance between 3D coordinates over the aortic wall, and lastly by calculating the circumferential apposition surface. The last 2 methods rely on dedicated postprocessing software. To date, large prospective trials are lacking to determine the best technique and associate post-EVAR complications with RASZ failure. Only small retrospective series have been published. Bastos Gonçalves and co-workers retrospectively studied the association between the early post-EVAR proximal seal and mid-term outcomes for the Excluder endoprosthesis.^15^ At a median follow-up of 4.1 years, a seal length <10 mm and the presence of an endoleak were significant risk factors for aneurysm-related adverse events in a relatively small cohort of 131 patients. In another retrospective study by Baderkhan et al, similar results were found.^16^ Main limitations included the relatively long median time to first post-EVAR CT angiography of 53 (0 – 355) days and a moderate degree of agreement for adequate classification into risk groups.

In the case of neck-related complications such as type Ia endoleaks, there was a strong consensus in the expert group to consider adjunctive procedures during EVAR. In general, type Ia endoleaks caused by too low initial deployment will be treated with a proximal cuff, whereas if the position of the proximal part of the endograft is adequate, reballooning or proximal adjunctive procedures (eg, endoanchors) might be considered. If a small, low flow type Ia endoleak persists despite good positioning and eventual adjunct procedures, the option to wait and see is considered valid, as these type Ia endoleaks may resolve spontaneously.^17^ In case of insufficient or suboptimal post-EVAR sealing zone without visible complications on the first postoperative CT, the consensus to perform adjunctive procedures was weak. In this case, some board members suggested a stricter CT follow-up protocol. Furthermore, one of the challenges during EVAR is the fact that the RASZ cannot be determined on the completion angiography. The only variable that can be measured is the distance from the lowest renal artery to the top of the endografts fabric, which must be related to the pre-EVAR measured neck length. The RASZ can only be measured on a CT scan, either performed at a hybrid operating theater or during post-EVAR follow-up.

An interesting finding is the strong consensus by the AB members to consider reintervention in case of negative evolution of the sealing zone over time (without complication), which would require at least 2 postoperative CT scans, perhaps even at predetermined moments. Again, literature to substantiate this outcome is sparse. Most of the publications regarding long-term EVAR outcomes focus on type Ia endoleaks, whether or not in combination with distal migration of endografts, and not on distal migration alone. If distal migration causes a type Ia endoleaks, it is advocated to reintervene because repressurization of the aneurysm increases the risk for rupture.^18,19^ If distal migration does not lead to type Ia endoleak, no clear cutoff has been defined yet whether, when, and how to intervene. Schuurmann et al determined changes in endograft position and proximal sealing zone post-EVAR and compared uncomplicated and complicated follow-up.^20^ One of the main conclusions by the authors was that progressive changes in endograft dimensions within the infrarenal neck could be detected on regular follow-up CT scans before the complication became urgent in many patients. Another important finding was that (subtle) decrease of the proximal sealing zone overtime was not only caused by distal migration of the endografts but also due to aortic neck dilatation. This last phenomenon leads to a loss of seal at the distal part of the sealing zone and will not be appreciated if CT scans are only judged for endograft migration during follow-up. Therefore, they advocate careful determination of post-EVAR sealing zone changes. Unfortunately, the authors did not advise any cutoff value for a minimum length of sealing zone, which is a limitation of their study.

Ideally, EVAR sizing and planning would evolve to a risk-stratified treatment and follow-up scheme based on patient demographics, TASZ geometry pre-EVAR, and RASZ post-EVAR. Currently, a possible method to anticipate the distal point of the TASZ is to use the nominal diameter of the main body of the endograft, which is selected during preoperative planning, and compare this with the aortic neck diameters. If the aortic neck diameter exceeds the nominal diameter of the endograft, the distal end of the TASZ can be defined. However, the interplay between the aortic geometry, aortic compliance, endograft oversizing, and device choice and positioning during implantation is very complex. Simplifying the aortic neck into essential geometrical components such as length, diameter, angulation, and conicity, which is currently the best practice, can result in unexpected TASZ loss. A solution could be provided by virtual stenting algorithms trained with various aortic shapes related to the RASZ and long-term durability of the seal. It is anticipated that such intelligent algorithms will better determine the TASZ, so it seems worthwhile to develop software for pre-EVAR virtual stenting.

Regardless of the preoperatively anticipated risk for failure of durable seal, it is crucial to monitor the result of the procedure. The positioning of the endograft and absence of type Ia endoleak can be verified during completion angiography, but the RASZ cannot. Intravascular ultrasound or cone-beam CT in a hybrid operating theater during the procedure may pose a solution. However, the added value for determining the RASZ intraoperatively instead of the 30 days CT scan has not been studied yet.

There was a strong consensus that the RASZ should be measured on the postoperative CT scans. Part of the AB members would intervene in case of short or suboptimal RASZ. A threshold for incomplete apposition has not yet been supported with clinical evidence, and therefore a deviation from the IFU (ie, <10 mm apposition) has been suggested as an alternative. Future studies should define risk-based cutoff values for insufficient RASZ and critical decrease of the RASZ.

### Limitations

During the selection of AB members, great effort was made to select vascular surgeons with extensive experience in EVAR and to represent the European geographical variety. By doing so, only vascular surgeons from large (academic) medical centers were selected. Therefore, the recommendations might not all be precisely applicable to other medical centers, especially outside Europe. This would largely depend on the expertise and facilities of those centers. Furthermore, in this Delphi consensus, the exact definitions of a “high degree of infrarenal angulation” and “challenging neck anatomy” were not defined, which could have influenced the respective answers by the AB members to these questions. Also, this Delphi consensus did not provide recommendations regarding the frequency of CT follow-up, which is especially interesting when the RASZ is suboptimal or decreasing at consecutive follow-up scans. This might be an interesting issue for future research. In addition, the current definition of RASZ, which was used in this Delphi consensus, did not consider an effective oversizing percentage of 10% to 15% in the infrarenal aortic neck, but was focused on the circumferential apposition to the aortic wall. In future research and consensus meetings, it is worthwhile to incorporate this refinement of the definition, as proper oversizing may be important to accomplish sustained seal during the entire cardiac cycle. This could be defined as real achieved effective sealing zone. Last, dedicated software to determine the TASZ and/or RASZ is being developed, but unfortunately, these are not yet Conformité Européenne (CE) -marked.

## Conclusion

While literature regarding sealing zone in EVAR patients is scarce, this study provides a broadly shared expert opinion based on the Delphi method. Advisory Board members agreed on important clinical definitions of TASZ and RASZ and their importance, and a recommendation for their measurement. Important anatomical, prosthetic, and procedural factors influencing the TASZ were established. Furthermore, AB members agreed on the necessity and timing of adjunctive procedure(s) and reintervention intraoperatively, directly postoperative, or during follow-up. By doing so, a clinical decision algorithm was proposed to aid physicians in their decision-making.
